# Spike patterning in oxytocin neurons: Capturing physiological behaviour with Hodgkin-Huxley and integrate-and-fire models

**DOI:** 10.1371/journal.pone.0180368

**Published:** 2017-07-06

**Authors:** Trystan Leng, Gareth Leng, Duncan J. MacGregor

**Affiliations:** Centre for Integrative Physiology, University of Edinburgh, Edinburgh, United Kingdom; SUNY Downstate MC, UNITED STATES

## Abstract

Integrate-and-fire (IF) models can provide close matches to the discharge activity of neurons, but do they oversimplify the biophysical properties of the neurons? A single compartment Hodgkin-Huxley (HH) model of the oxytocin neuron has previously been developed, incorporating biophysical measurements of channel properties obtained in vitro. A simpler modified integrate-and-fire model has also been developed, which can match well the characteristic spike patterning of oxytocin neurons as observed *in vivo*. Here, we extended the HH model to incorporate synaptic input, to enable us to compare spike activity in the model with experimental data obtained *in vivo*. We refined the HH model parameters to closely match the data, and then matched the same experimental data with a modified IF model, using an evolutionary algorithm to optimise parameter matching. Finally we compared the properties of the modified HH model with those of the IF model to seek an explanation for differences between spike patterning *in vitro* and *in vivo*. We show that, with slight modifications, the original HH model, like the IF model, is able to closely match both the interspike interval (ISI) distributions of oxytocin neurons and the observed variability of spike firing rates *in vivo* and *in vitro*. This close match of both models to data depends on the presence of a slow activity-dependent hyperpolarisation (AHP); this is represented in both models and the parameters used in the HH model representation match well with optimal parameters of the IF model found by an evolutionary algorithm. The ability of both models to fit data closely also depends on a shorter hyperpolarising after potential (HAP); this is explicitly represented in the IF model, but in the HH model, it emerges from a combination of several components. The critical elements of this combination are identified.

## Introduction

The supraoptic nucleus of the hypothalamus has been a rich source of insight into how the intrinsic properties of neurons are adapted to meet physiological requirements. It contains only neuroendocrine neurons that secrete their peptide products into the circulation from nerve terminals in the posterior pituitary gland. Some of these neurons make vasopressin, which acts at the kidneys and the peripheral vasculature to mediate antidiuresis and to control plasma volume, the rest make oxytocin, which promotes uterine contractions during parturition and mediates milk let-down in response to suckling. However, both oxytocin and vasopressin have additional roles, some mediated by release of oxytocin and vasopressin within the brain, and some by actions at other peripheral targets [1,2]. These two populations display very different discharge characteristics, and extensive studies *in vitro* have characterised their intrinsic membrane properties [3–7], while studies *in vivo* have characterised their responses to physiological challenges [1,8]. During suckling in lactating rats, oxytocin neurons discharge in intermittent bursts that give rise to pulses of oxytocin secretion. The same neurons, in response to increases in plasma osmotic pressure, show graded increases in electrical activity [9] that result in increases in plasma oxytocin that modulate sodium excretion by actions at the heart and kidneys. Oxytocin neurons are osmosensitive: the increases in osmotic pressure result in a graded depolarisation of membrane potential, and, in addition, they receive synaptic input from other osmosensitive neurons in anterior brain regions [10,11].

In oxytocin neurons, spike patterning modulates the response to input signals and also determines the Ca^2+^ entry that triggers exocytosis from axonal terminals. Typically, clustering of spikes facilitates this; Ca^2+^ entry is coupled non-linearly to spike activity by complex mechanisms at the terminals [12,13]. The patterns in which oxytocin neurons fire are strongly influenced by two intrinsic activity-dependent mechanisms: Each spike is followed by a hyperpolarising afterpotential (HAP) that makes the neuron relatively inexcitable for up to 100 ms, and also by a much slower activity-dependent afterhyperpolarisation (AHP). Following individual spikes, the AHP is very small, but after a high frequency burst of spikes, the AHP forms a conspicuous long hyperpolarisation.

*In vivo*, data from extracellular single-cell recordings comprise series of spike times. These can be used to analyse spike patterning, using techniques such as inter-spike interval (ISI) distributions, hazard functions, and index of dispersion (IoD) (also known as ‘Fano Factor’) [14,15]. Although we can’t use *in vivo* data to identify the underlying mechanisms, we can detect and quantify their effect. Looking at the distribution of ISIs allows us to detect and measure features such as the HAP. Analysis over multiple ISIs, using IoD to examine the timescale dependence of ISI variability, allows us to detect more subtle features such as the AHP. Using integrate-and-fire (IF) based modelling we can simulate simplified versions of these afterpotentials to generate spike times and apply the same ISI analysis techniques, matching the model output closely to experimental data. Our modified adaptive IF model removes the post-spike reset of the classic IF model, and uses spike-incremented, exponentially decaying variables to represent post-spike hyperpolarisations and depolarisations, like the spike-response model [16], but retaining a continuous differential-equation based form.

In an IF model with just an HAP [9] we previously concluded that, for an increase in synaptic activity to produce a linear increase in spike rate, the input must comprise a mixture of EPSPs and IPSPs. We tested this experimentally, and confirmed that osmotic stimulation indeed increases the release of both the excitatory neurotransmitter glutamate and the inhibitory neurotransmitter GABA in the supraoptic nucleus.

In non-lactating rats the oxytocin neurons do not communicate directly with each other, but they do in lactating rats, and milk-ejection bursts arise as a result of interactions between the oxytocin neurons [17]. Using a network of IF model neurons to simulate the bursting, we concluded that the AHP is important in “shaping” the bursts. However, the AHP also affects discharge patterning in non-lactating animals at low spike rates, acting as a slow negative feedback that regularises the firing rate over a time course of seconds. We showed in the IF model that the variability of spike activity is markedly reduced by the AHP [15].

Thus IF models can provide close matches to the spike activity of oxytocin neurons, and relate intrinsic properties such as the afterpotentials to function, but do these models oversimplify the biophysical properties of the neurons?

Fitting the IF model to *in vivo* data has helped us to match patterning features detected in ISI analysis to these afterpotentials, and also quantify their magnitude and time course. To test these matches, or to identify which afterpotentials might exist, we need to be able to relate the simplified afterpotentials to the ionic currents that shape neuronal membrane activity. *In vitro*, we lose most of the synaptic inputs that stimulate the neurons, but have much more control over the input signal, allowing the detection and measurement of the properties of the ionic currents. These can be assembled to build a detailed simulation of membrane activity using Hodgkin-Huxley (HH) style modelling. An existing oxytocin neuron HH model [18] models eight ionic currents, subsets of which act to generate a HAP and an AHP. The AHP is attributed to the Ca^2+^-dependent SK-type K^+^ current, but the HAP is more complex, thought to be the summed action of the delayed rectifier (KDR), the A-type K^+^, and the BK-type K^+^ currents, combining both voltage and Ca^2+^ dependence, on multiple time courses.

The afterpotentials therefore have multiple identities, as their effect on spike patterning, as their simplified forms in the IF model, and as the complex action of ionic currents. The same is true of other intrinsic, pattern influencing, properties of neurons, such as the K^+^ leak current which is thought responsible for phasic firing in vasopressin neurons [19–21] in its guise as a slow DAP. Here, we are attempting to map between these identities in oxytocin neurons, using modelling to bridge *in vivo* and *in vitro* experimental data.

Our aim in the present study was thus to relate the simple IF model of the oxytocin neuron to the more complex HH model. Our approach is to apply both the IF and HH oxytocin models to the same set of *in vivo* data, comparing the parameters and model elements which are associated with fitting the various spike patterning features. This is straightforward with the IF model, and to produce an objective fit, we have developed a new automated fitting process, based on a genetic algorithm. However, the HH model has only previously been tested against *in vitro* data and requires the addition of synaptic input to simulate *in vivo* data. Neurons *in vitro* are usually stimulated either by an applied current or by a depolarising solution, replacing the *in vivo* noisy synaptic input signal with a fixed depolarisation. This results in very different spike patterning *in vitro*, and it is not clear whether this is purely due to the change in input signal, or because the loss of synaptic input, and the resulting change in membrane properties, significantly alters the properties of the ionic currents.

## Methods

Supraoptic oxytocin neurons in virgin rats *in vitro* have a mean (SD) resting potential of −62 (7.2) mV [22], and spikes are triggered when depolarising current drives the membrane above a threshold of ∼ -50 mV. The spikes (measured from within 5 mV of spike threshold) have a mean (SD) amplitude of 73.2 (7.6) mV and a width at half-maximal amplitude of 1.5 (0.4) ms. The repolarization phase overshoots rest and merges into an HAP, which lasts for up to ∼ 100 ms [23] with a maximum magnitude of 8.6 (5.7) mV [24]. Trains of spikes evoke an AHP with a peak amplitude of 0.9 (0.3) mV per spike, which decays with a time constant of 520 (567) ms [24]. The AHP is abolished by removing extracellular Ca^2+^, and is attenuated by exposure to apamin [24–26], which selectively blocks SK-type small conductance K^+^ channels.

In the supraoptic nucleus, glutamate is the predominant excitatory, and GABA the predominant inhibitory neurotransmitter [27], each accounting for about a third of all synaptic input. EPSPs typically elevate the membrane potential by a few mV when the cell is at rest. The density of synapses in the supraoptic nucleus is about 35 x 10^6^ per mm^3^ of tissue [28]; the volume of the nucleus is about 2 mm^3^ (2 x 1 x 1 mm) and it contains about 4500 neurons [29]. The nucleus only contains magnocellular neurons, about a third of which are oxytocin neurons and the remainder vasopressin neurons, so assuming that these are equally densely innervated, there are about 15000 synapses for each neuron. Cells receive synaptic inputs from diverse sites including afferent forebrain regions such as the subfornical organ and organum vasculosum of the lamina terminalis, the caudal brainstem, the arcuate nucleus, nucleus accumbens, and from the perinuclear zone dorsal to the nucleus [1]. *In vitro*, most of these inputs are missing and most neurons have truncated dendrites; observed EPSP and IPSP rates are low, and random, spontaneous GABAergic synaptic activity (mostly from miniature synaptic potentials) dominates 4–5 fold over excitatory activity. Accordingly, most neurons are inactive unless depolarised to close to spike threshold. *In vivo*, under urethane anesthesia, oxytocin neurons are active at 2–5 spikes/s in basal conditions [15], but the synaptic input rate is not known.

### The IF model

We used an IF model described in [15,21]. The model simulates the firing response to randomly timed, exponentially decaying inputs, representing EPSPs and IPSPs at mean rates *I*_re_ and *I*_ri_, where *I*_ri_ is defined as a proportion of *I*_re_ given by *I*_ratio_. We assumed that EPSPs and IPSPs have equal and opposite magnitude (*e*_h_ = 3 mV and *i*_h_ = -3 mV) and a half-life (λ_syn_) of 8 ms; the precise values of these do not have a critical impact on any of the conclusions that we come to below, within a range of physiologically plausible values. *V*_syn_ represents the summed EPSPs and IPSPs. Other model variables represent the HAP and the AHP. After a spike, *HAP* and *AHP* are incremented by fixed values *k*_HAP_, and *k*_AHP_, and these potentials decay exponentially with half-lives λ_HAP,_ and λ_AHP_. In contrast to the classic IF model, there is no post-spike reset of the variables, allowing *AHP* to accumulate across multiple spikes. The model variables are summed with the resting potential (*V*_rest_, here fixed at -66 mV to match the HH model) to generate a membrane potential *V* where

$$V=V_{\text{rest}}+V_{\text{syn}}-HAP-AHP$$

When *V* exceeds the spike threshold (*V*_thresh_, fixed at -48 mV), the neuron fires a spike and the spike time is recorded. The large, fast decaying *HAP* produces a post-spike relative refractory period. The model uses a 1-ms step size, with simulations run for 1000–10000 s of simulated activity. The default IF model parameters, chosen to match the equivalent physiological parameters of the HH model, are given in Table 1.

**Table 1:** IF model default parameters.

Name	Description	Value	Units
*I*_re_	excitatory input rate	600	Hz
*I*_ratio_	inhibitory input ratio	0.5	-
*e*_h_	EPSP amplitude	3	mV
*i*_h_	IPSP amplitude	-3	mV
λ_*syn*_	PSP half life	8	ms
*k*_*HAP*_	HAP amplitude per spike	60	mV
λ_*HAP*_	HAP half life	8	ms
*k*_*AHP*_	AHP amplitude per spike	0.5	mV
λ_*AHP*_	AHP half life	500	ms
*V*_rest_	resting potential	-66	mV
*V*_thresh_	spike threshold potential	-48	mV

### Evolutionary parameter fitting

We used a genetic algorithm (GA) to automate parameter fitting with the IF model. This searches the parameter space by randomly generating a population of parameter sets, here called ‘chromes’, using these to run the model, and calculating a measure of fit for each chrome by comparing the model-generated spike times with those from a recorded neuron. The best chromes are then interbred, applying random crossover and mutation of their parameters, to make a new generation. After enough generations, the algorithm should converge on a parameter set which closely fits the target data.

We used a 128 chrome population, varying parameters *k*_HAP_, *k*_AHP_, λ_HAP,_ λ_AHP_, and *I*_re_, with initial values randomly generated from the ranges specified in Table 2, chosen to go well above and below the normal range for these parameters, balancing flexible search with physiologically plausible values. The range for *k*_AHP_ starts at 0, allowing fits with no AHP. All other parameters were fixed as in Table 1.

**Table 2:** IF model: Genetic algorithm fit parameters.

Name	Min Value	Max Value	Fit Value
*I*_re_	50	5000	334 (2.3), 648 (9)
*k*_*HAP*_	10	500	83
λ_*HAP*_	2	50	8
*k*_*AHP*_	0	5	0.77
λ_*AHP*_	50	1500	482

We ran each chrome to generate 1000-s trains of spikes and for each generated a fit score to the *in vivo* reference data, using four analysis measures generated for both the *in vivo* and model generated spike times; the front (first 30 bins) and tail (bins 30 to 125) of the ISI distribution, the hazard function (Sabatier et al. 2004), and the IoD for bin sizes of 0.5,1, 2, 4 and 8 s (Royo *et al*. 2016), calculating the fit score for each using a root mean square (RMS) error measure. The ISI distribution, generated from limited data with uniform 5-ms bin sizes, has the problem of being noisy and highly skewed, with a fit measure that gives equal weight to each bin. To fix this, we use an ISI distribution (and hazard function generated from this) with histogram bin sizes which increase in size on an exponential scale, generated based on the quadratic formula:

$$\text{bin}=\left(-0.975+\sqrt{(0.975^{2}+0.1\text{ISI}})\right)/0.05\;(\mathrm{bin}\;\mathrm{is}\;\mathrm{quantized}\;\mathrm{to}\;\mathrm{the}\;\mathrm{nearest}\;\mathrm{integer})$$

The distribution is also smoothed using a five bin window. The code, with full details of the fit measures and RMS error calculations, is given in the supplementary material (S1 Code). The four RMS scores were weighted, summed, and scaled to generate a single fit score using the formula:

$$\mathrm{fit}\;\mathrm{score}=\left(\mathrm{fron}t_{\mathrm{RMS}}\;\mathrm{fron}t_{\mathrm{Weight}}+\mathrm{tai}l_{\mathrm{RMS}}\;\mathrm{tai}l_{\mathrm{Weight}}+\mathrm{ha}z_{\mathrm{RMS}}\;\mathrm{ha}z_{\mathrm{Weight}}+\mathrm{Io}D_{\mathrm{RMS}}\;\mathrm{Io}D_{\mathrm{Weight}}\right)/\left(\mathrm{fron}t_{\mathrm{Weight}}+\mathrm{tai}l_{\mathrm{Weight}}+\mathrm{ha}z_{\mathrm{Weight}}+\mathrm{Io}D_{\mathrm{Weight}}\right)$$

The 32 chromes with the best (lowest) fit scores were then interbred to generate a new 128 chrome generation, using two-point crossover and mutation. Full mutation (randomly generating an entire new chrome independent of parents) occurs with 0.05 probability. Otherwise, two parents are randomly selected (from the 32) and a new chrome is generated using crossover, where two random points define a section of the parameters copied from one parent, and the rest from the other. Each parameter also has mutation applied in the form of a random offset proportional to the difference in value for that parameter between the two parents. The next generation’s parents are then selected from the best of the 32 parent chromes and the 128 new chromes, so that parents are only discarded when improved upon. The code, with full details, is given in the supplementary material (S1 Code). We ran the genetic algorithm for 20 generations, and took the result as the best chrome from the final generation.

The implementation is mainly in C++ but uses CUDA [30] based code to run the IF model in parallel for the whole population on a GPU. A run of 20 generations with a population of 128 takes ~17 s on an Nvidia GTX 960 graphics card.

### The HH model

The original oxytocin HH model that we began with has been described fully elsewhere [18], so here we give an abbreviated account. We renamed currents *I*_KDR_ (previously *I*_K_ or *I*_DR_), *I*_BK_ (previously *I*_C_), and *I*_SK_ (previously *I*_AHP_). We also made some corrections to the published equations, based on model code kindly supplied by Peter Roper (detailed below).

The model is a single compartment, with electrical activity modelled as:

$$\frac{dV}{dt}=-\frac{1}{C}\left(I_{\text{Na}}+I_{\text{Ca}}+I_{\text{KDR}}+I_{\text{A}}+I_{\text{BK}}+I_{\text{SK}}+I_{\text{SOR}}+I_{leak}\right)$$

All voltage- and Ca^2+^-dependent currents have the standard activation/inactivation form:

$$I_{\gamma }(t)=g_{\gamma }m_{\gamma }^{\alpha }(t)h_{\gamma }^{\beta }(t)(V-E_{rev})\;\text{or}\;I_{\gamma }(t)=g_{\gamma }m_{\gamma }^{\alpha }(t)(V-E_{rev})$$

The first equation describes an inactivating current, with gating variables *m*_γ_ (activation) and *h*_γ_ (inactivation), and the second a non-inactivating current with just *m*_γ_. Each activation (*m*) or inactivation (*h*) gating variable *x*(*t*) evolves to an equilibrium *x*_∞_ with time constant *τ*_*x*_, according to:

$$\frac{d}{dt}x(t)=\frac{x_{\infty }-x(t)}{\tau_{x}}$$

Each current *I*_γ_ has conductance *g*_γ_ and reversal potential *E*_rev_. The Na^+^ and K^+^ reversal potentials are *E*_Na_ = 50 mV and *E*_K_ = -96 mV. The Ca^2+^ reversal potential is given by:

$$E_{\text{Ca}}=12.5\log_{\text{n}}\left(\frac{{\left[{\text{Ca}}^{2+}\right]}_{\text{o}}}{{\left[{\text{Ca}}^{2+}\right]}_{\text{i}}}\right)$$

where [Ca^2+^]_o_ = 4 mM, and [Ca^2+^]_i_, varies as *C*_i_, described below. We assumed a capacitance *C* = τ*/R* of 1 *μ*F/cm^2^ [31].

***I***_**Na**_ is a fast Na^+^ current [32] which mediates the upstroke of the spike, and has a cubic activation and linear inactivation. We assume that it activates instantaneously (*m*(*t*) = *m*_∞_).
***I***_**Ca**_ is a high-voltage activated non-inactivating Ca^2+^ current. Supraoptic neurons express several high- and intermediate-voltage activated Ca^2+^ channels; these are active only during a spike, and so are approximated by a single current. *Correction to the Roper et al 2003 model description*: *I*_Ca_(*t*) = *g*_Ca_*m*^2^(*V*−*E*_Ca_) (*m* instead of *m*_∞_)
***I***_**KDR**_ is the delayed rectifier voltage-sensitive K^+^ current [33].
***I***_**A**_ is a transient A-type inactivating voltage sensitive K^+^ current [33,34].
***I***_**SK**_ is a Ca^2+^-dependent K^+^ current carried by small conductance (SK) channels [25,35,26], and is controlled by Ca^2+^ in a micro-domain close to the membrane. *I*_SK_ underlies spike frequency adaptation during repetitive firing and the AHP. *Correction to the Roper et al 2003 model description*:
  $$q_{\infty }(C_{\text{SK}})={\left(1+\exp\left[-1.120-2.508\log_{10}\left(\frac{C_{\text{SK}}-C_{\text{r}}}{1000}\right)\right]\right)}^{-1}\;(\log_{10}\;\mathrm{instead}\;\mathrm{of}\;\log)$$
***I***_**BK**_ is a Ca^2+^- and voltage-dependent K^+^ current carried by BK channels [36].
***I***_**SOR**_ is the sustained outward-rectifier K^+^ current that is present in oxytocin neurons but not in vasopressin neurons [3]: it activates slowly, and only when the cell is close to spike threshold, and is non-inactivating.
***I***_**leak**_ is a leak current which models processes that comprise the passive response of the membrane and accounts for the membrane time constant (12–16 ms; [24]). It comprises Na^+^ and K^+^ components:
  $$I_{\text{leak}}=g_{\text{Naleak}}(V-E_{\text{Na}})+g_{\text{Kleak}}(V-E_{\text{K}})$$
  where *g*_Naleak_ = 0.018 and g_Kleak_ = 0.066, generating a leak reversal potential of ~65mV.

### Intracellular Ca^2+^

To simulate the microdomains created by the proximity of *I*_BK_ and *I*_SK_’s Ca^2+^ sensors to membrane Ca^2+^ channels, the model has two channel-specific [Ca^2+^]_i_ compartments, *C*_BK_, *C*_SK_, as well as the bulk compartment, *C*_i_. The BK channels are close to the Ca^2+^ channels and experience a large, fast-changing Ca^2+^ signal. The SK channels are further away, and experience a smaller, slower changing signal. Ca^2+^ concentrations evolve according to:

$$\frac{d}{dt}C_{\gamma }=-\alpha_{\gamma }I_{\text{Ca}}-\frac{1}{\tau_{\gamma }}(C_{\gamma }-C_{r})$$

where γ = {BK, SK, i}. *α*_*γ*_ relates the amplitude of the Ca^2+^ current to Ca^2+^ influx. The time constant τ_γ_ approximates the processes that return the Ca^2+^ transient to rest. *C*_r_ is the basal Ca^2+^ concentration; the mean value observed experimentally is 113 nM, and spike-induced increases decline exponentially with a mean (SD) time constant of 0.99 (1.6) s [18].

### Synaptic input

We model synaptic input as a random train of excitatory and inhibitory post-synaptic currents (EPSCs and IPSCs):

$$I_{\text{syn}}=m_{\text{ipsc}}(V-E_{\text{ipsc}})+m_{\text{epsc}}(V-E_{\text{epsc}})$$

The same Poisson processes as used in the IF model generate EPSC and IPSC counts *n*_epsc_ and *n*_ipsc_ at each time step, at mean rates *R*_epsc_ and *R*_ipsc_. The gating variables *m*_epsc_ and *m*_ipsc_ describe the summation and decay of the synaptic events:

$$\frac{d}{dt}m_{\gamma }=-\frac{m_{\gamma }}{\tau_{\gamma }}+\Delta_{\gamma }n_{\gamma }$$

where γ = {epsc, ipsc}. Each PSC is modelled as an instantaneous rise of amplitude Δ_γ_ followed by an exponential decay with time constant 3.3 ms for both EPSCs (τ_epsc_) and IPSCs (τ_ipsc_).

### ISI distributions

The ISI distributions are constructed as histograms using 5-ms bins, and normalised to 10000 ISIs.

### Implementation

Both models were implemented using software developed in C++ with the open source wxWidgets graphical interface library [37]. The HH model differential equations were run using the Runge-Kutta method, with a 50-μs time step. A single run of the HH model, simulating 1000 s of activity takes ~43 s. on an Intel i7-5960X processor running at 3.5GHz. An equivalent run of the IF model takes ~0.3 s, more than 100 times faster. The HH model is much slower both because of its much larger set of equations, and its heavy use of compute-intensive math functions. Profiling using the open source CPU profiling software ‘Very Sleepy CS’ shows that compute time running the HH model is dominated by the ‘exp’ and ‘pow’ functions.

## Results

### Reference data

**Fig 1:**
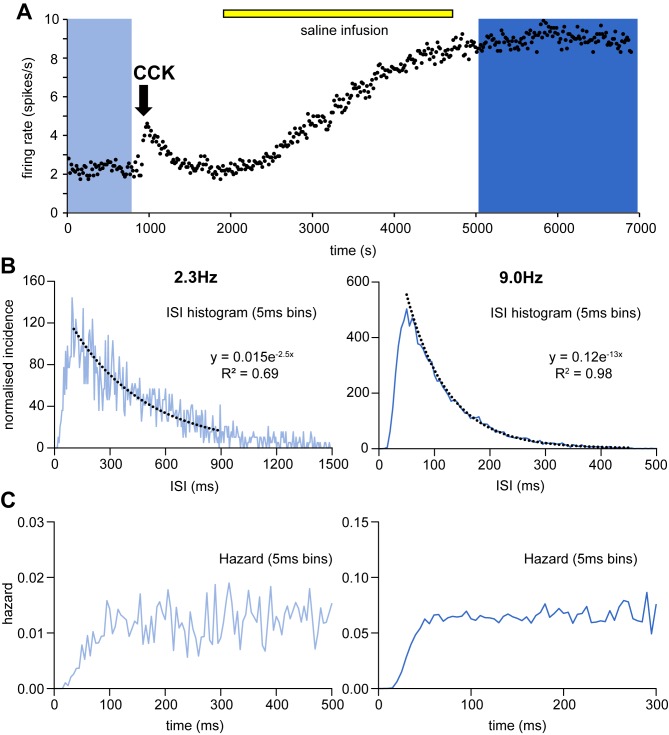
Reference data for model fitting. **(A**) shows the mean firing rate of an oxytocin neuron (in 16-s bins) recorded from the supraoptic nucleus of a urethane-anesthetised rat. The neuron was antidromically identified as projecting to the posterior pituitary gland and identified as an oxytocin neuron by the transient excitation in response to CCK (arrow). The neuron was recorded throughout an i.v. infusion of hypertonic saline (yellow bar) which increased its firing rate from 2.3 spikes/s to 9.0 spikes/s. See [9] for full experimental details. **(B)** shows on the left, the ISI distribution for the 850-s period of low frequency activity (1945 spikes) indicated by the light blue background, and on the right, the ISI distribution for the 2000-s period of high frequency activity (17236 spikes) indicated by the dark blue background (S1 Data). These distributions are typical of oxytocin cells, and have been normalised to 10000 ISIs. The ISI distributions after the mode are well fit by a negative exponential (black dotted lines, equations of best fit given). Fewer spikes produces a noisier distribution. **(C)** shows the hazard functions (in the same 5-ms bins) corresponding to the ISI distributions in **B**. A flat hazard indicates no time dependent influence on the chance of firing. The initial climb from 0 shows the effect of the HAP.

From a large library of recordings of oxytocin neurons *in vivo*, we selected one long recording for matching to the models (Fig 1). The chosen recording is from a single supraoptic neuron of an adult virgin female rat anaesthetised with urethane (ethyl carbamate, 1.3 g/kg body weight i.p.) in which the supraoptic nucleus and neural stalk were exposed by ventral surgery and a femoral vein was cannulated for i.v injection of cholecystokinin (CCK). The neuron was antidromically identified as projecting to the neural stalk to identify it as a magnocellular neurosecretory neuron, and it was identified as an oxytocin neuron by its excitatory response to CCK (see [38]. The cell was also exposed to an i.v. infusion of hypertonic saline, which increased its firing rate linearly from ~2.3 to ~9 spikes/s. This recording was chosen because its spontaneous discharge characteristics and its responses to CCK [15,38] and hypertonic saline [9] are all typical of oxytocin neurons *in vivo*. At 9 spikes/s, the mode of the ISI distribution is well defined at ~ 55 ms, close to the mean (50 ms; SD 13 ms) ISI mode for oxytocin cells [38]. The ISI distribution is skewed: at 2.3 spikes/s, just 1.3% of ISIs are < 55 ms (Fig 1B). At 9 spikes/s, 17.5% of ISIs are shorter than the mode, and the distribution of ISIs longer than the mode is well fit by a single negative exponential (Fig 1B). This cell did not exhibit an apparent DAP as inferred from hazard functions (Fig 1C); a fast DAP is present in a minority of oxytocin cells [15,39] but is not included in the models described here: the biophysical basis of the fast DAP is not yet defined.

### Adding synaptic input to the HH model

**Fig 2:**
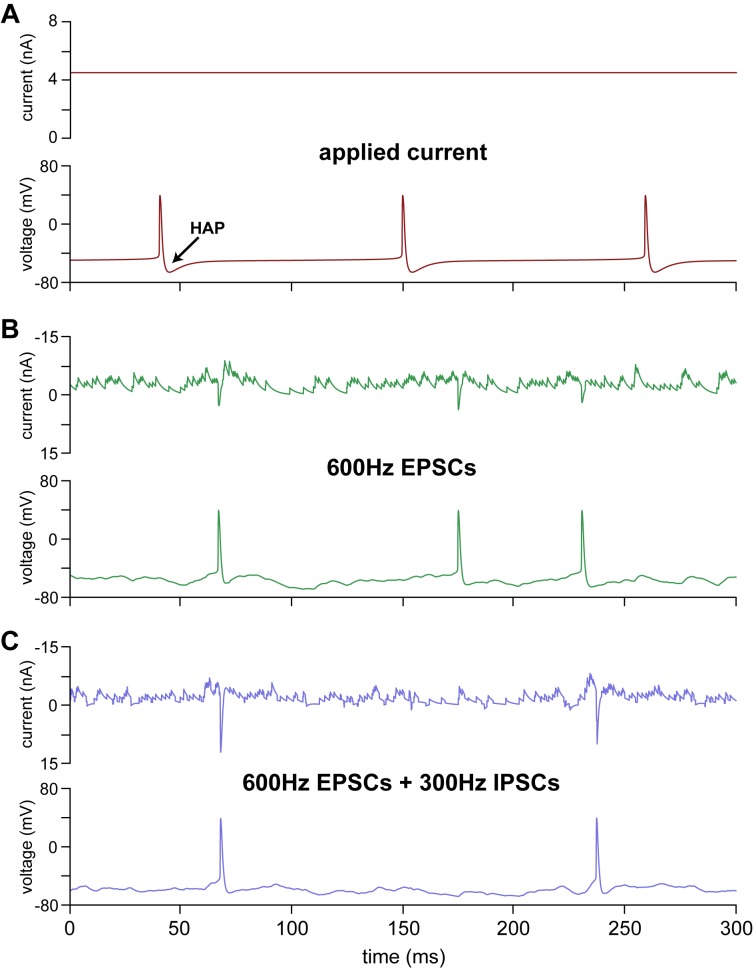
Adding synaptic input to the oxytocin neuron HH model. **(A)** The original oxytocin HH model, challenged with a constant applied current of 4.5 nA produces regular firing at 10 spikes/s; each spike is followed by a conspicuous but short HAP. **(B)** shows the HH model with EPSCs at 600 Hz (*R*_epsc_ = 600, *R*_ipsc_ = 0), generating spikes at a mean rate of 5.6 spikes/s. The randomly timed EPSCs generate a noisy spiking membrane potential that much more closely resembles an *in vivo* neuron than the regular spikes produced by an applied current. Note that the post-spike HAP is less conspicuous and of more varied appearance than in **A**. **(C)** shows the HH model with EPSCs at 600 Hz, and IPSCs at 300 Hz (*R*_epsc_ = 600, *R*_ipsc_ = 300), generating spikes at a mean rate of 1.6 spikes/s. Note that the HAP following spikes is even less conspicuous than in **B**.

The original HH model [18] has a resting potential of -65.7 mV and a spike threshold of -47.8 mV, and, in response to an applied current, it produces regularly timed spikes of stereotypical form (Fig 2A). To test it against *in vivo* data, we added synaptic input (EPSCs and IPSCs, Fig 2B and 2C), using synaptic parameters (Table 3) that produced EPSP and IPSPs of 2 to 3 mV at a typical inter-spike membrane potential, ~58 mV. From a resting potential of -65.7 mV, an EPSC amplitude (Δ_epsc_) of 0.024 produced an EPSP of 3 mV, and an IPSC amplitude (Δ_ipsc_) of 0.059 produced an IPSP of 1 mV (the amplitudes also depend on the time constants *τ*_epsc_ and *τ*_ipsc_, both set at 3.3 ms). The IPSC reversal potential, *E*_ipsc_ = -75 mV makes IPSCs more sensitive to membrane potential during subthreshold activity than EPSCs, so typical IPSPs are larger than the 1 mV seen at rest. The decay phases of EPSPs and IPSPs are mainly determined by the membrane time constant (~12 ms), which is governed by *I*_leak_. The inputs generate a noisy, irregularly spiking, membrane potential that resembles an *in vivo* neuron [40,41] much more closely than the regularly-timed spikes produced by the applied current (Fig 2A). With EPSCs at 600 Hz, the HH model generates 5.6 spikes/s (Fig 2B), and adding IPSCs at 300 Hz reduces the firing rate to 1.6 spikes/s (Fig 2C).

**Table 3:** HH model synaptic input parameters.

Name	Description	Value	Units
*R*_epsc_	excitatory input rate	0 to 10000	Hz
*I*_ratio_	inhibitory input ratio	0 to 0.5	-
Δ_epsc_	EPSC amplitude	0.024	nA
Δ_ipsc_	IPSC amplitude	0.059	nA
τ_epsc_	EPSC decay time constant	3.3	ms
τ_ipsc_	IPSC decay time constant	3.3	ms
*E*_epsc_	EPSC reversal potential	0	mV
*E*_ipsc_	IPSC reversal potential	-75	mV

### Fitting the IF model

We fixed the IF model parameters *V*_rest_ = -66 mV and *V*_thresh_ = -48 mV to match the measured membrane properties of the HH model, and fixed the synaptic parameters λ_syn_ = 8 ms, *e*_h_ = 3 mV, and *i*_h_ = -3 mV to match the HH model’s PSP magnitudes and decay rates.

**Fig 3:**
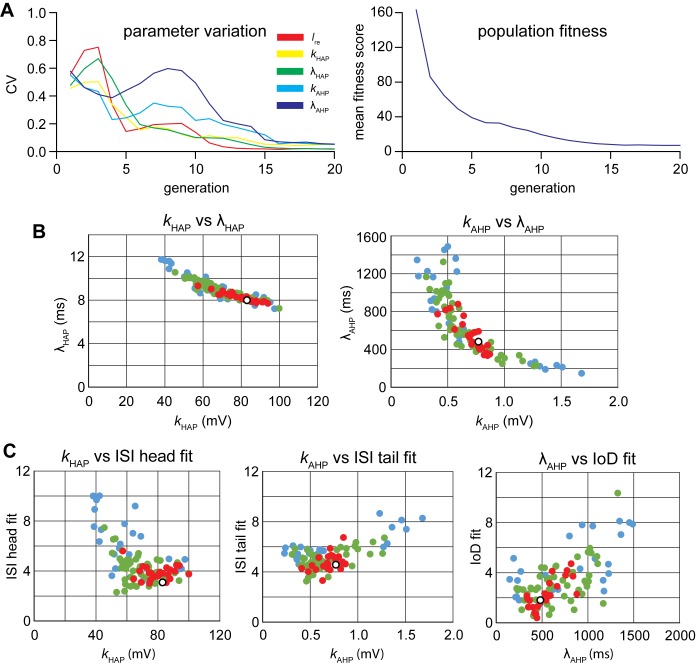
Fitting the IF model using a genetic algorithm. A genetic algorithm was used to find values for five parameters of the IF model that give an optimal fit to the reference data of Fig 1. **(A)** shows how a single run of the algorithm (128 parameter sets or ‘chromes’) converges over 20 generations. The left panel shows the five parameters, plotting the coefficient of variation (CV) for each parameter across the population. The right panel shows mean population fitness improving. The best fit parameters and fitness score (range 3.69 to 7.38) varied between runs. **(B)** shows that the values found for the half-life of the HAP (λ_HAP_) are inversely related to the values found for the magnitude of the HAP (*k*_HAP_), and that the values found for the half-life of the AHP (λ_AHP_) are inversely related to the values found for the magnitude of the AHP (*k*_AHP_): thus these parameters are not independent, but compensate against each other to some extent. (**C)** plots found parameters against relevant elements of the fit measure. The plots in C and D are colour coded by overall fit measure. Red shows the top 25%, green 25% to 50%, and blue the bottom 25%. The outliers tend to have poorer fit scores, whereas the red values are mostly clustered in a small range. To choose a single best fit we took the median value for parameters from the 10 best fits, shown by the white dots, which each fall within the red clusters. The final parameters (for the 9 spikes/s data) are *I*_re_ = 648 Hz, *k*_HAP_ = 83 mV, λ_HAP_ = 8 ms, *k*_AHP_ = 0.77, and λ_AHP_ = 482 ms.

To fit the IF model to the *in vivo* reference data, we developed an automated, genetic algorithm (GA) similar to that which we previously used to fit a vasopressin neuron IF model [42], and fitted the remaining parameters to match the data for the period in which the reference neuron was firing at 9 spikes/s (Fig 1). We fitted five parameters: the HAP magnitude and half-life (*k*_HAP_ and λ_HAP_), the AHP magnitude and half-life (*k*_AHP_ and λ_AHP_), and the synaptic input rate (*I*_re_). We ran the algorithm for 20 generations with a population of 128 parameter sets (‘chromes’). This was sufficient for the population to converge (Fig 3A), and the final fit for each run was chosen as the chrome in the final generation with the best fit.

**Fig 4:**
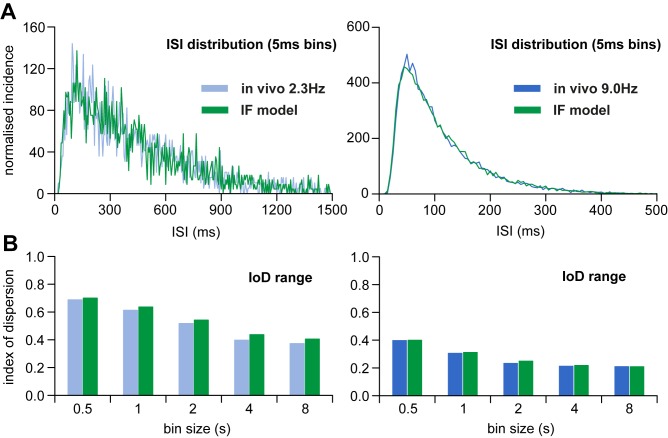
The IF model genetic algorithm fitted to *in vivo* oxytocin data. **(A)** shows in blue the ISI distribution in the reference data (Fig 1) for spike activity at 2.3 spikes/s (left) and 9 spikes/s (right), and in green the fit achieved with the parameter set found by the genetic algorithm (Fig 3). We used the parameters given in Fig 3 to generate the fit to the data at 9 spikes/s; to fit the 2.3 spikes/s data we reran the genetic algorithm with all parameters fixed except the synaptic input rate (*I*_re_). This achieved a good fit with *I*_re_ = 334 Hz. **(B)** shows the corresponding matches to IoD values (reference data in blue, model data in green).

The parameters of the best fits varied between runs. To explore this, we ran the genetic algorithm 100 times, and plotted the final parameters against each other (Fig 3B), and against relevant elements of the fit measure (Fig 3C). The parameters of the best fits are not independent; for both the HAP and the AHP, the parameters for magnitude and half-life were inversely related (Fig 3B). The best fits were mostly clustered in a small range, and to choose a single best fit we took the median value for each parameter from the 10 best fits: *I*_re_ = 648 Hz, *k*_HAP_ = 83 mV, λ_HAP_ = 8 ms, *k*_AHP_ = 0.77, and λ_AHP_ = 482 ms. We then tested this parameter set against the 2.3 spikes/s data from the same cell (Fig 1A), running the algorithm with all parameters fixed except *I*_re_, achieving a good fit with *I*_re_ = 334 Hz (Fig 4).

The parameters found are consistent with previous fits to the HAP and AHP [15], with an AHP that most closely matches the time course of the apamin-sensitive medium AHP [25]. We have no good experimental evidence for the true proportion of excitatory and inhibitory synaptic input *in vivo*, so we repeated the fitting for different balances of excitatory and inhibitory synaptic input (*I*_ratio_ = 0, 0.5, and 1), but found no consistent difference in fit scores or fitted parameters. For subsequent simulations and the results shown here we used *I*_ratio_ = 0.5.

### Fitting the HH model: The HAP and the ISI distribution

**Fig 5:**
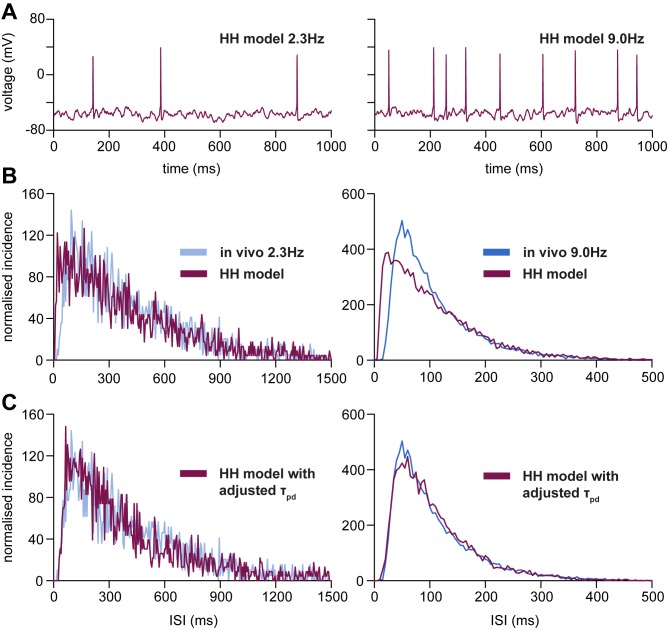
Fitting the HH model to ISI distributions. (A) shows examples of the HH model spiking activity used to generate the ISI distributions. **(B)** shows in maroon the ISI distributions generated by the original HH model, at firing rates of 2.3 spikes/s (left) and 9 spikes/s (right), compared with reference data (blue). These were achieved with EPSC rates (*R*_epsc_) of 505 Hz and 769 Hz. In both the proportion of short ISIs is larger than in the reference data (in blue), and the mode is earlier. **(C)** shows the ISI distributions from the HH model with τ_pd_, the deactivation time constant of *I*_BK_, increased from 1.22 to 10 ms. EPSC rates (*R*_epsc_) were slightly increased, to 506 Hz and 774 Hz, to reacquire the firing rates of 2.3 and 9 spikes/s. In the adjusted model, the ISI distributions fit the reference distributions well at both firing rates.

The original oxytocin HH model was used to generate 1000-s simulated recordings at ~2.3 and ~9 spikes/s (Fig 5A) for comparison with the two regions of steady firing rate in the reference data (Fig 1A). We challenged the model with EPSCs at various rates to find values of *R*_epsc_ for which the model spike rate matched the target firing rate. A spike rate of 2.3 spikes/s was produced with *R*_epsc_ = 505 Hz, and 9 spikes/s with *R*_epsc_ = 769 Hz. At both rates, the model generated an excess of short ISIs (< 50 ms), with an earlier mode of the ISI distribution (25 vs 50 ms at 9 spikes/s; Fig 5B), indicating a weaker HAP than observed in the reference data.

In the HH model, the HAP is generated by the combined effect of *I*_KDR_, *I*_A_, and *I*_BK_ [18], but the sustained component is thought to be due to *I*_BK_ [43]. Removing *I*_A_ (by setting *g*_A_ = 0) increased the mean firing rate (from 2.3 to 3.8 spikes/s and from 9 to 10.5 spikes/s) with no effect on the mode of the ISI distribution. Removing *I*_KDR_ (by setting *g*_KDR_ = 0) decreased the firing rate (from 2.3 to 2.1 spikes/s and from 9 to 8.5 spikes/s), but again the shape of the ISI distribution was unaffected. *I*_BK_ is both voltage and Ca^2+^ sensitive, and a key parameter determining the HAP is the time constant τ_p_, set at 1.22 ms. This short time constant is required for *I*_BK_ to activate during a spike, but for the HAP to be sustained, *I*_BK_ must deactivate more slowly. The time-constant τ_p_ of this channel was originally set at 1.22 ms for both activation and deactivation; we explored the effects of increasing the time-constant independently for deactivation, replacing τ_p_ with τ_pa_ (activation) and τ_pd_ (deactivation).

Studies of supraoptic neurons *in vitro* [36] concluded that the open time constant for the BK channel is ~8.5 ms, while closed times are best fitted by two exponentials with time constants of 1.6 and 12.7 ms. We therefore increased τ_pd_ to 5, 10, and 15 ms, adjusting *R*_epsc_ to generate trains at 9 spikes/s. Increasing τ_pd_ decreased the proportion of short ISIs, increased the height of the ISI mode, and shifted the mode to the right, and with τ_pd_ at 10 ms we could closely match the target ISI distributions at both firing rates (Fig 5C). At 9 spikes/s (*R*_epsc_ = 774 Hz), 18.3% of ISIs were < 55 ms (compared to 17.5% in the reference data; Fig 1B) and the mode was ~60 ms; at 2.3 spikes/s, 2.0% of ISIs were < 55 ms (1.3% in the reference data; Fig 1B). In ten simulations with different random sequences of PSCs, the mean (SD) intervals < 55 ms at 2.3 spikes/s was 2.2 (0.2) % (range 1.7–2.5%), and at 9 spikes/s was 18.8 (0.3) % (range 18.2–19.2%). Hence, the increased τ_pd_ consistently produced a closer match to the reference ISI distributions at both 2.3 spikes/s (*R*_epsc_ = 506 Hz) and 9 spikes/s (*R*_epsc_ = 774 Hz; Fig 5C). The 10 ms deactivation time constant is similar to the fitted λ_HAP_ (8 ms half-life = 11.5 ms time constant) in the IF model, suggesting that the HAP in the IF model captures the effect of prolonged BK channel activation.

### The BK current and the HAP

**Fig 6:**
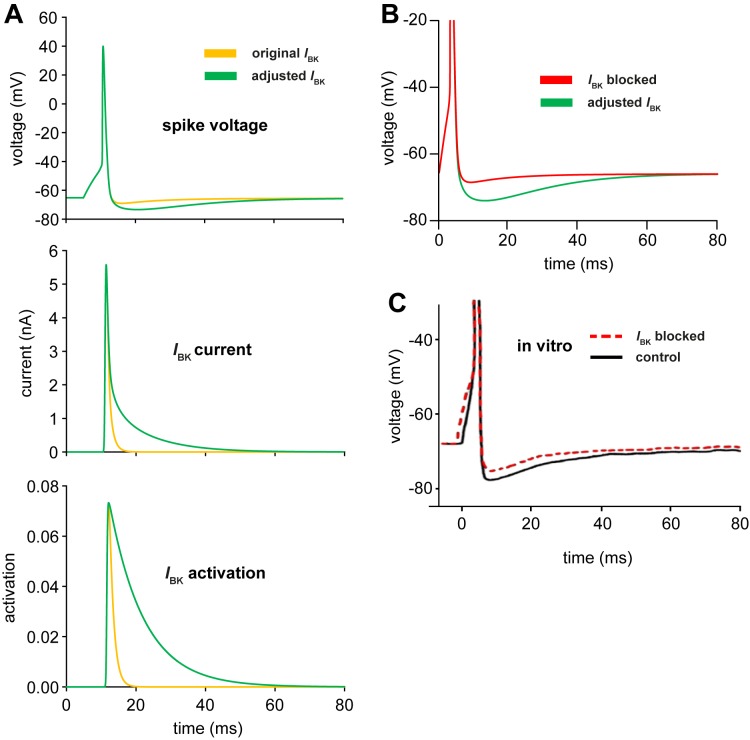
Spike waveforms in the HH model with varied BK. **(A)** shows the voltage, *I*_BK_ current, and *I*_BK_ activation behaviour of a spike induced by a 5-nA current step for 5 ms, in the original oxytocin HH model (yellow, τ_pd_ = 1.22 ms), and the adjusted model (green; τ_pd_ = 10 ms). Experimentally, the HAP lasts for 25–125 ms and hyperpolarises the cell by ~7.5 mV [18]. In the original HH model, the HAP is too short and too small, because *I*_BK_ both rises and falls sharply after a spike. In the adjusted model, *I*_BK_ is prolonged by the slower deactivation, making a better match to the HAP observed *in vitro*, as well as improving the fit to the *in vivo* ISI distribution. **(B)** shows, for the adjusted model, the effects of setting *g*_BK_ = 0 (red, compared to original value *g*_BK_ = 1, in green), on a single spike induced by an 8-nA current step for 3 ms, to simulate the pharmacological block of *I*_BK_ [18]. When *I*_BK_ is blocked, the HAP is smaller and decays faster. **(C)** shows the *in vitro* experiment data simulated in (B), redrawn from [18]. The duration and amplitude of the HAP is a much closer match to the adjusted model. The block shows only a partial reduction, but is likely to be less total than the model.

To understand what effect the slower deactivation of *I*_BK_ has on the HAP, we studied the changes during a single spike (Fig 6A). Increasing the deactivation time constant τ_pd_ from 1.22 to 10 ms increased the active duration of *I*_BK_ from ~10 to ~50 ms. The 50 ms corresponds to the duration of the apparent refractory period in the reference ISI distribution (Fig 1B). Blocking *I*_BK_ by setting *g*_BK_ = 0 left only a small, residual HAP due to *I*_A_ and *I*_KDR_ (Fig 6B). The equivalent experimental data (Fig 6C) where *I*_BK_ is blocked *in vitro* using iberiotoxin [18] also shows a reduction in the HAP; the reduction is smaller than in the model, but the pharmacological block is likely to be less complete. The *in vitro* data also shows an HAP duration of ~50 ms, so the adjusted model gives a better fit to both *in vivo* and *in vitro* data.

### The effects of synaptic input properties

**Fig 7:**
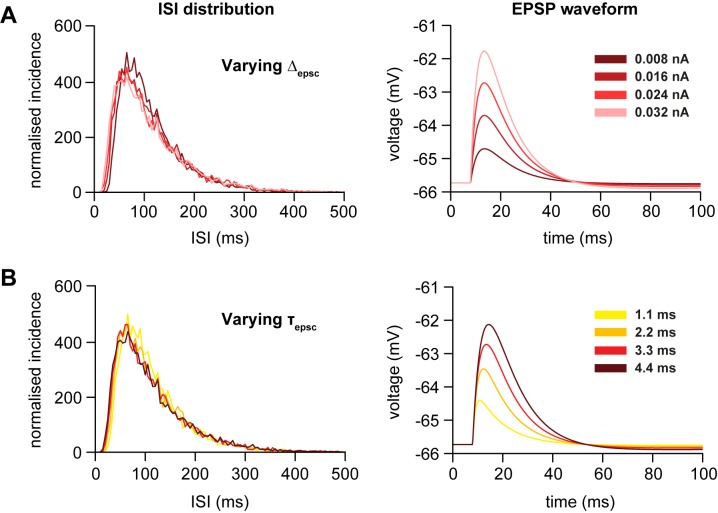
Changes in EPSC parameters have little effect on the ISI distribution. We investigated how changes to the EPSC parameters affect the ISI distribution in the HH model when the spike rate is kept constant at 9 spikes/s. (**A)** Increasing the EPSC magnitude (Δ_epsc_) slightly increases the number of short ISIs, shifting the distribution to the left, with a small decrease in the height of the mode. Plotted distributions are for EPSP magnitudes of ~ 1, 2, 3, and 4 mV (darkest to lightest). The changes in EPSPs that result from the changes in *k*_epsc_ are plotted on the right. EPSC rates (*R*_epsc_ = 2920, 1294, 774 and 526 Hz respectively) were adjusted to achieve a firing rate of 9 spikes/s. **(B)** Increasing the EPSC time-constant (τ_epsc_) very slightly increases the number of short ISIs, shifting the distribution to the left, with a small decrease in the height of the mode. Plotted distributions are for τ_epsc_ = 1.1, 2.2, 3.3, and 4.4 ms (yellow to dark red). The changes in EPSPs that result from the changes in τ_epsc_ are plotted on the right. EPSC rates (*R*_epsc_ = 2685, 1220, 774, and 567 Hz respectively) were adjusted to achieve a firing rate of 9 spikes/s.

The IF model is relatively insensitive to changes in synaptic input parameters, in that changes in PSP decay rates and magnitudes have effects that can be mimicked simply by changing the input rates. We asked how changes to synaptic input parameters in the HH model affect the ISI distribution. With τ_pd_ at 10 ms, the model was used to generate spikes at 9 spikes/s with varied Δ_epsc_ and τ_epsc_, adjusting *R*_epsc_ to match the 9 spikes/s firing rate. Increasing EPSC amplitude (changing Δ_epsc_ from 0.008 to 0.032 nA to increase EPSPs from ~1 to 4 mV) slightly shifted the ISI distribution towards shorter ISIs but with no change in mode (Fig 7A). Increasing τ_epsc_ had a similar effect; an increase from 1.1 to 4.4 ms produced a small shift in the ISI distribution with no change in mode (Fig 7B). Thus EPSC amplitudes and decay rates had little effect on the ISI distribution (for a given firing rate) over a wide range of physiologically plausible values.

### Adding inhibitory synaptic input

In previous work fitting oxytocin neurons with the IF model we showed that an equal balance of EPSPs and IPSPs (*I*_ratio_ = 1) increased the linearity of the spike response [9]. In an IF model with fixed magnitude PSPs it is easy to produce an exact balance of inhibitory and excitatory input, but this is not practicable in a HH model with reversal potentials, where PSP magnitudes shift in opposite directions with increased membrane activity. Testing balanced EPSC and IPSC rates (*I*_ratio_ = 1), the HH model is unable to fire faster than 3 spikes/s: the output response peaks at a ~ 3000 Hz input rate, before slowly declining with increased input (not shown). The competing IPSPs increase in magnitude as well as rate, creating a non-linear increase in the inhibitory component of the synaptic signal and maintaining a membrane potential that is generally too hyperpolarised to trigger spiking. Reducing *I*_ratio_ to 0.5 allowed spiking at 10 spikes/s with a 2000 Hz input rate (*R*_epsc_ = 2000).

**Fig 8:**
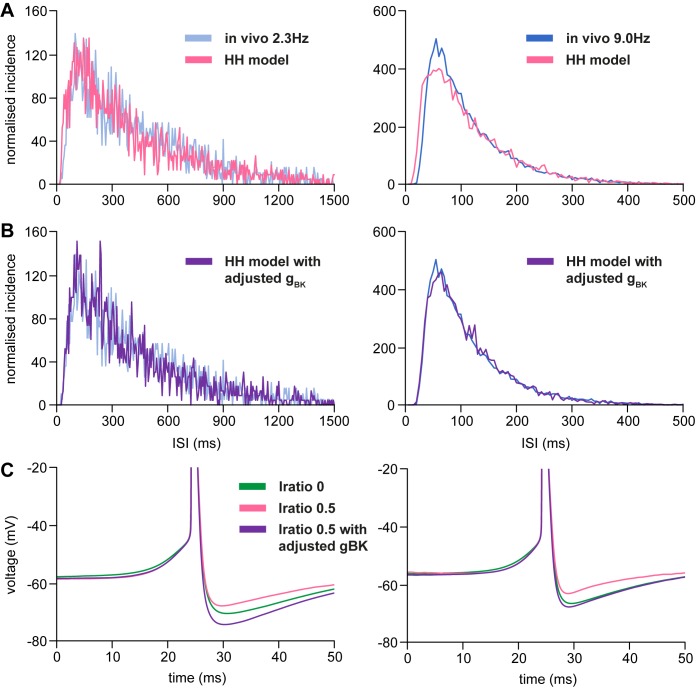
Effects of adding inhibitory synaptic input. **(A)** shows the effect of increasing *I*_ratio_ to 0.5 from 0 on the ISI distributions generated by the HH model at 2.3 and 9 spikes/s (*R*_epsc_ was adjusted to 694 and 1304 Hz to achieve firing rates of 2.3 and 9 spikes). Model distributions (pink; *I*_ratio_ = 0.5, *g*_BK_ = 1) are superimposed on ISI distributions from the reference data in Fig 1 (blue). Increasing *I*_ratio_ increases the number of short ISIs, and the model ISI distribution no longer matches the reference data. **(B)** shows the effect of increasing *g*_BK_ to 3.2 mS/cm^2^, with *R*_epsc_ adjusted to 694 and 1290 Hz to achieve firing rates of 2.3 and 9 spikes/s. ISI distributions from the adjusted model (purple; *I*_ratio_ = 0.5, *g*_BK_ = 3.2) are superimposed on ISI distributions from the reference data in Fig 1 (blue). By increasing *g*_BK_, a good fit can again be obtained. **(C)** shows the ‘average spike waveform’ for three different runs of the model–*I*_ratio_ = 0 in green (from Fig 6B), *I*_ratio_ = 0.5, *g*_BK_ = 1 in pink, and *I*_ratio_ = 0.5, *g*_BK_ = 3.2 in purple. Increasing *I*_*ratio*_ results in an attenuation of the observed HAP, which can be reversed by increasing *g*_BK_, restoring a good fit to the *in vivo* reference data.

We studied whether introducing IPSCs has any effect on spike activity beyond requiring a higher rate of EPSCs to produce a given firing rate. Increasing *I*_ratio_ from 0 to 0.5 (while adjusting the overall input rate to match the target spike rate) increased the proportion of short ISIs at both 2.3 and 9 spikes/s, shifting the ISI distribution to the left (Fig 8A). To understand why, we reconstructed ‘average spike waveforms’ (Fig 8C). In the presence of IPSCs (*I*_ratio_ = 0.5) the HAP is greatly attenuated. The shape of the HAP could be restored by increasing the conductance of the BK channel (*g*_BK_) from 1 to 3.2, and this also restored the fit to the ISI distribution (Fig 8B).

Increasing *I*_ratio_ without also increasing the EPSC rate showed no attenuation of the HAP in the spike waveform (not shown), indicating that the reduced HAP magnitude is due to the increased rate of EPSCs. The increased rate of IPSCs fails to counter this effect due to the HAP taking the membrane very close to the IPSC reversal potential at -75mV.

### Spike rate variability and the AHP

**Fig 9:**
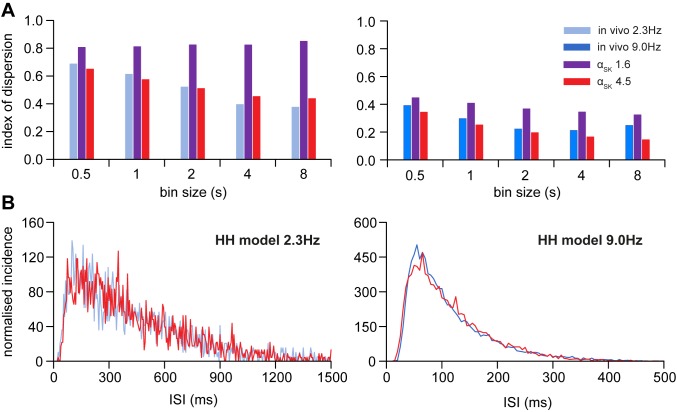
Fitting the HH model to the IoD range. The IoD measured at a range of selected binwidths shows the timescale dependence of spike rate variability. **(A)** compares the IoD range of the reference data, at 2.3 (light blue) and 9 spikes/s (light blue), with two variations of the HH model. Data from the model of Fig 8 are in purple. The data in red are from the model with stronger Ca^2+^ activation of the AHP generating SK channel (*α*_SK_ changed from 1.6 to 4.5). The EPSC rate (*R*_epsc_) was increased to 746 and 1860 Hz to achieve firing rates of 2.3 and 9 spikes/s with *I*_ratio_ = 0.5. The previous (Fig 8) version of the HH produces IoD values that are too high, implying that spike events are more variable than found experimentally. In the model with *α*_SK_ increased to 4.5, the IoD values closely match the reference data at both spike rates, suggesting that the AHP plays a large role in spike rate variability in an oxytocin cell. (**B)** compares the ISI distributions of the model with *α*_SK_ = 4.5 to the reference data. With the changes to the AHP, the model ISI distributions still fit experimental data well at both firing rates. Thus, for a given firing rate, the parameters of the AHP current have little effect on the ISI distribution.

The AHP, which is small but long-lasting, has little effect on the ISI distribution [14] but a large effect on the variability of firing rate [15]. This variability can be captured by the IoD of the firing rate. Randomly timed spikes, uninfluenced by previous activity, give an IoD value of 1 that is independent of firing rate and binwidth. The *in vivo* reference data at both 2.3 and 9 spikes/s show IoD values well below 1 (Fig 9A), and which decrease as binwidth increases up to 4 s, indicating that some activity-dependent influence reduces the variability of firing rate. Previous work with the IF model showed that this was due to the AHP [15].

We ran the HH model for 1000 s, with *τ*_pd_ = 10 ms, *I*_ratio_ = 0.5, *g*_BK_ = 3.2 (as in Fig 8B). The IoD values at both 2.3 spikes/s (*R*_epsc_ = 694 Hz) and 9 spikes/s (*R*_epsc_ = 1290 Hz) were higher than in the reference data, and at 2.3 spikes/s showed no dependence on binwidth. This indicated that the AHP generated by the SK current in the HH model was too small. The SK current is purely Ca^2+^ dependent, and, to strengthen the activity dependence of the AHP, we increased α_SK_ (the scaling factor which relates the amplitude of the Ca^2+^ current (*I*_Ca_) to the SK specific Ca^2+^ concentration) from 1.6 to 4.5, increasing *R*_epsc_ to 746 Hz and 1860 Hz to match 2.3 and 9 spikes/s respectively. With these changes, the IoD values closely matched the reference data (Fig 9A), and the ISI distributions still fitted the reference data well at both firing rates (Fig 9B). The final adjusted parameters are given in Table 4. Thus, for a given firing rate, strengthening the AHP current strongly reduces spike rate variability, while having little effect on the ISI distribution.

**Table 4:** Adjusted HH model parameters.

Name	Original	Adjusted
τ_pd_	1.22 ms	10 ms
*g*_BK_	1	3.2 mS cm^-2^
α_SK_	1.6	4.5

### Input balance and spike rate response

**Fig 10:**
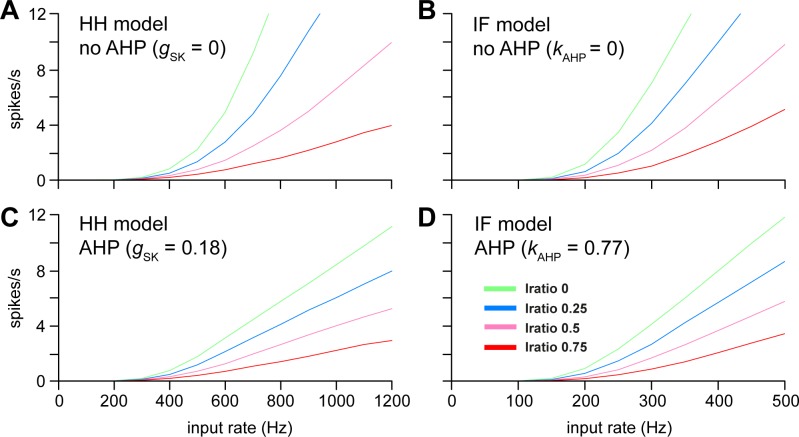
Spike rate response with varied *I*_ratio_ and AHP. The panels show how the spiking rate increases with the synaptic input rate (increasing *R*_EPSC_ with varied *I*_ratio_ = 0, 0.25, 0.5 and 0.75), comparing the HH model (left) and IF model (right). **(A)** With no AHP (*g*_SK_ = 0), the HH model with only excitatory input (*I*_ratio_ = 0) shows a highly non-linear response, which becomes increasingly linear as *I*_*r*atio_ is increased. **(B)** shows a similar result with the genetic algorithm-fitted IF model with *k*_AHP_ = 0, matching [9]. **(C)** With the AHP (*g*_SK_ = 0.18) the HH model shows a more linear response even with *I*_*r*atio_ = 0. **(D)** Similarly in the IF model, the AHP linearises the response to increasing input. Thus, the AHP, in both the HH and IF models, has a similar linearising effect to increasing the ratio of inhibitory input.

In an IF model with no AHP, adding inhibitory synaptic input linearizes the response to increasing synaptic input [9]. Using our final version of the HH model, we plotted the relationship between input (PSC) frequency (parameter *R*_epsc_) and spike rate for different values of *I*_ratio_ and tested the effect of removing the AHP by setting *g*_SK_ = 0. With *I*_ratio_ = 0 (i.e. with EPSCs only) and no AHP, spike output increases non-linearly (Fig 10A). The physiological range of spike rates (0–12 spikes/s) is encompassed by an approximate doubling of EPSC frequency (from ~350 to ~ 700 Hz). Increasing *I*_ratio_ reduces the slope of the relationship between input rate and output rate without altering the threshold at which spike activity starts to increase, and linearizes the input-output response throughout the physiological range of firing rates. For *I*_ratio_ = 0.5, the physiological range of spike rates (0–12 spikes/s) is encompassed by increasing EPSC frequency (from ~ 350 to ~ 2400 Hz). This result with the HH model matches the previous IF model result, reproduced with the current IF model in Fig 10B.

However, comparing models with and without an AHP showed that the AHP itself has a strong linearizing effect. With *I*_ratio_ = 0 and an AHP, spike output increases non-linearly up to about 2 spikes/s as EPSC frequency increases, but above this the increase is approximately linear with EPSC frequency (Fig 10C). In all conditions, the same tests with the IF model (Fig 10B and 10D) showed very similar results, except that a lower input rate was required to match a given spike rate. PSP magnitude in the IF model was fixed to match PSP magnitude in the HH model at resting potential, but in the HH model, due to reversal potentials, EPSPs become smaller and IPSPs become larger with increased activity, and resulting in a higher PSP rate required to produce the same firing rate.

### Matching spike patterning in vitro

**Fig 11:**
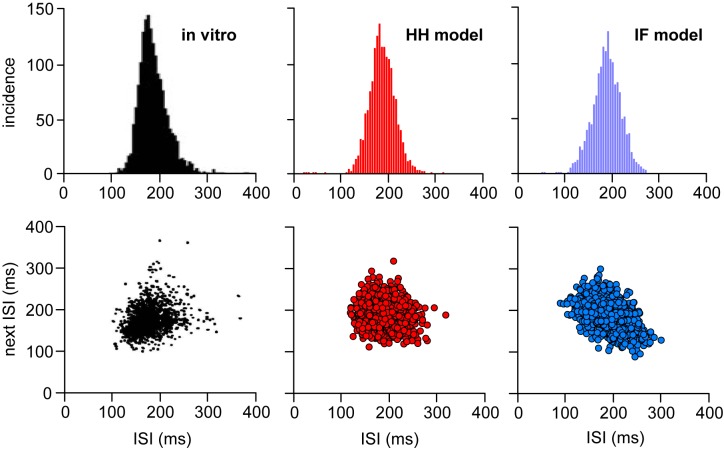
Matching ISI distributions from oxytocin neurons *in vitro*. Neurons recorded *in vitro* (redrawn from [6]), stimulated by a constant depolarising current, show a much narrower ISI distribution, indicating a more regular spiking rate, much closer to a normal distribution than observed *in vivo* (Fig 1). The remaining variability can be attributed to membrane noise, or some low level of residual synaptic input. Using the same HH and IF models, with parameters fitted to the *in vivo* reference data, we were able to match the *in vitro* ISI distribution using a constant applied input combined with a low rate of low magnitude excitatory synaptic input. In the HH model (red) we used a 4.5 nA constant input with EPSC parameters Δ_epsc_ = 0.008 and *R*_epsc_ = 120 Hz, producing 5.3 spikes/s. In the IF model we used *V*_ext_ = 20.3 mV with EPSP parameters *I*_re_ = 120 Hz and *e*_h_ = 0.08 mV, and *I*_ratio_ = 0. The cluster plots indicate that ISIs are independent of the preceding ISI.

Oxytocin neurons *in vivo* and *in vitro* show very different ISI distributions [38]. Spiking *in vitro* (in explant or slice preparations) is mostly stimulated by a constant applied input, or by exposure to a depolarising solution, with little residual synaptic input, and it shows a narrow, Gaussian ISI distribution [6] (Fig 11) rather than the long-tailed Poissonian distribution observed *in vivo* (Fig 1B). We previously suggested that the differences were due to changes in membrane properties caused by the loss of synaptic input, resulting in changes to the currents that determine spike patterning. However, by adding small EPSCs at a low frequency to simulate membrane noise and residual synaptic activity (Δ_epsc_ = 0.008, *R*_epsc_ = 120 Hz, *I*_*ratio*_ = 0, with 4.5 nA current step), we were able to closely match the *in vitro* ISI distribution using the same HH model parameters that fit the *in vivo* data. This suggests that the differences between the spike patterning *in vivo* and *in vitro* can be explained purely by the change in input signal. We reproduced the same result in the IF model (see Methods) (Fig 11) by adding constant *V*_ext_ to simulate an applied voltage:

$$V=V_{\text{rest}}+V_{\text{syn}}-HAP-AHP+V_{\text{ext}}$$

where *V*_ext_ = 20.3 mV, with synaptic input parameters *I*_re_ = 120 Hz, *e*_h_ = 0.08 mV, and *I*_ratio_ = 0. The low value synaptic input parameters are used to simulate residual synaptic activity, most of the input signal is due to the applied voltage *V*_ext_, equivalent to the current step in the HH model.

## Discussion

The common advantage of HH models and IF models is their ‘observability’, the ability to relate their parameters to measurable physiological values [44]. This study has shown that a HH model of oxytocin neurons, based on current dynamics and spike waveforms derived from *in vitro* experiments, with minor modifications and the addition of a simulated random synaptic input, can fit the ISI distribution of spike times and other statistical features of spike patterning recorded *in vivo*. This allows a direct comparison with an IF model in terms of how well they match functionally relevant data, and enables a better understanding of how the simplified HAP and AHP in the IF model relate to detailed current-based mechanisms.

The ISI distributions of oxytocin neurons *in vivo* and *in vitro* are very different [38]. As well as the loss of a noisy input signal, the loss of synaptic input *in vitro* changes a cell’s input resistance, and we had assumed that this would alter current dynamics, particularly voltage sensitivity. We had therefore expected that, because the HH model of [18] was based on *in vitro* data, we would need to modify many of its parameters to fit *in vivo* data. However, the HH model, when stimulated with a randomly timed sequence of EPSCs, produced an ISI distribution that was a surprisingly close match to our *in vivo* reference data. It showed an excess of short ISIs, but this was corrected by modifying just one parameter, increasing the BK current deactivation time constant to increase HAP duration.

The HAP has a dual identity as *mechanism* and *effect*. It is the summed action of K^+^ channels activated after a spike, and can be observed both as a prolonged hyperpolarisation in the spike waveform and as a shortage of short ISIs in the ISI distribution. The dominant element of the mechanism is *I*_BK_, a voltage and Ca^2+^-dependent K^+^ current carried by BK channels. In the original model, activation and deactivation shared a single time constant, and the requirement for rapid voltage-sensitive activation during a spike had dictated its small value. Increasing the deactivation time constant from 1.2 ms to 10 ms was sufficient to achieve a close match between model and *in vivo* ISI distributions, and is consistent with *in vitro* data studying BK channel kinetics [36], as well as making a better match to the post-spike waveform (Fig 6).

The HAP in the IF model uses a much simpler mechanism of a two-parameter (magnitude and decay rate) post-spike decaying exponential. It requires a larger magnitude (~60 mV) to match the same refractory effect of the HAP in the HH model (~15 mV), but its GA-fitted time constant (11.5 ms) is close to the HH model’s BK deactivation. The IF model’s HAP can thus be considered as a representation of the HH model’s HAP effect on excitability, rather than its effect on membrane potential.

A second discrepancy between the HH model and *in vivo* observations emerged when comparing the variability of spike rates measured using IoD over a range of binwidths. The firing rate of oxytocin neurons is more regular when averaged in bins of a few seconds than expected from the variability of ISIs, and in an IF model, this can be explained by the presence of an AHP [15]. The AHP cannot be detected in the spike waveform or the ISI distribution, but it can be observed using analysis which is sensitive to effects over multiple ISIs. We showed here, in the HH model, that increasing the duration and/or amplitude of the AHP similarly results in a close match to experimental measurements of IoD, and does so without degrading the ability to match the shape of the ISI distribution.

The simpler, purely Ca^2+^-dependent mechanism of the AHP allows a more direct translation between the HH and IF models. The AHP is generated by a Ca^2+^-dependent K^+^ current carried by SK channels. The HH model’s SK channel specific Ca^2+^ variable, *C*_SK,_ subject to a sigmoidal activation function, directly drives *I*_SK_. *C*_SK_ increases with the transitory peaks of *I*_Ca_ corresponding to each spike, and decays exponentially with time constant 656 ms, following a very similar form to the spike-incremented exponentially decaying AHP variable in the IF model, with a GA-fitted decay time constant of 695 ms (half-life 482 ms).

Thus, with slight adjustments, the HH model closely fits the spontaneous spike discharge activity of oxytocin neurons *in vivo*, as measured by spike rate, ISI distribution and IoD range. We cannot claim that it will fit all oxytocin cells, as we matched the model to just a single representative example, and some oxytocin neurons have a DAP that is not represented in this model. However, in oxytocin neurons that lack a DAP, the variation in statistical properties can be matched in IF models by relatively slight variations in the HAP (or, in some cases, by variations in the proportion of IPSCs and EPSCs) and by variations in the AHP, and by equivalent variations in the HH model.

The IF model can be fit objectively by an evolutionary algorithm, an approach that we used previously to model vasopressin cells [42]. We have previously fitted the oxytocin IF model by sequential adjustments of parameters [14,15], but the lack of independence of the parameters and the use of multiple fit measures (spike rate, ISI distribution, and IoD range) makes this time consuming, and it is less objective than using an automated method. An objective fit is especially important for making a comparison with the HH model where we can directly compare parameter values. Although results varied between runs, the clustering in parameter space of the best fit scores indicates that the fit measure is sufficiently robust to choose a single best fit. The speed of a highly parallel GPU based implementation makes multiple runs very practical.

The present analysis has forced refinement of other conclusions from our previous work. Our original IF model [9] included an HAP but not an AHP. That model was matched to ISI distributions alone: the AHP has little effect on these, and its importance only becomes apparent from higher order statistical features of spike patterning. In that paper, we reported that oxytocin neurons increase their firing rate linearly when challenged with a linear increase in osmotic pressure, as illustrated by the representative recording chosen for the present work (Fig 1). This was difficult to reconcile with the non-linearity of stimulus-response characteristics of the simple IF model, but we noted that if the model was challenged not with a linear increase of EPSP frequency but with a linear increase of a balanced mixture of EPSPs and IPSPs, then the response was linear. We then showed, by measuring release of GABA and glutamate in the supraoptic nucleus, that osmotic challenge was indeed accompanied by release of both inhibitory and excitatory neurotransmitters. However, we show here that, for both the modified HH model and the modified IF model, the AHP can linearize the stimulus-response characteristics over much of the physiological dynamic range, without requiring IPSPs. Thus, we cannot infer from the linearity of response to osmotic pressure that this response *necessarily* involves both inhibitory and excitatory components, although experimental data indicate that it does.

So is there any feature of the spike activity of oxytocin neurons from which we might infer the relative balance of inhibitory and excitatory inputs that they are receiving? This balance does affect statistical features of spike activity: for any given firing rate, a higher proportion of IPSCs in the input, balanced by a higher rate of EPSCs, will result in more short ISIs. This is a consequence of the effect of reversal potentials: during a post-spike hyperpolarisation, IPSCs (which reverse at -75 mV) will be smaller, while EPSCs (which reverse at 0 mV) will be larger, resulting in an HAP which is functionally shortened. Thus, paradoxically, a synaptic input signal with an increased proportion of inhibitory inputs favours the generation of short ISIs, that are likely, because of the frequency dependent non-linearity of stimulus secretion coupling at nerve terminals, to result in facilitated transmitter release at nerve endings.

The ideal neuronal model is one which matches the function of real neurons, is computationally efficient, and which can be easily matched to experimental data, both in its parameters and output. HH models are valued because they more completely model the mechanisms underlying neuronal excitability, with the assumption of a more complete functional match to real neuron properties. They are also a good match to the bottom-up approach and data available from studying neurons *in vitro*. They are computationally expensive and potentially difficult to apply to neurons *in vivo*, though we have shown that this is not a difficult leap if *in vitro* data is available. In comparison, basic IF models are more analytically tractable and computationally efficient, at the expense of simplified functional behaviour. Our style of modified IF models trades some of the analytical tractability for a more complete functional match by adding post-spike potentials such as the HAP and AHP, and we have shown here that their simplified representations are a good match to the more complex equivalent mechanisms in the HH model. [45] has even argued that the threshold triggered spiking of the IF model is actually a better match to the highly non-linear spike initiation dynamics of real neurons than the HH model’s mechanism. The best approach might be to work between the two, using HH modelling to map detailed *in vitro* experimental data on individual currents to functional elements. These can be added in simplified form to an IF model, modelling greater scales of time or numbers of neurons in a system which is both more intuitive and faster to run.

## Supporting information

S1 Code HH and IF models and genetic algorithm source code. HH model, IF model, and genetic algorithm fitting C++, IF model CUDA source code.
Files: oxymodbasic.h, oxymodbasic.cpp, oxygenbasic.cpp, evofitbasic.h, evofitbasic.cpp, evofitscore.cpp, evodat.h, evodat.cpp, evospikegen.cu.
(ZIP)

S1 Data Oxytocin neuron *in vivo* spike times. 9 spikes/s and 2.3 spikes/s spike time (ms) data.
Files: oxy9hz.txt, oxy2-3hz.txt.
(ZIP)
